# Natural products for intervertebral disc degeneration: mechanistic insights and therapeutic potentials

**DOI:** 10.3389/fphar.2025.1605764

**Published:** 2025-07-25

**Authors:** Zenghan Wu, Jiang Chen, Wenhai Luo, Tao Kuang

**Affiliations:** ^1^ The First Hospital of Hunan University of Chinese Medicine, Changsha, China; ^2^ Ningbo Chinese Medicine Hospital, Ningbo, Zhejiang, China

**Keywords:** natural products, IDD (intervertebral disc degeneration), mechanistic, hyperoside, quercetin

## Abstract

Intervertebral disc degeneration (IDD) is a leading cause of spinal disorders worldwide. Current clinical therapies for IDD are often constrained by limited efficacy, notable adverse effects, and high treatment costs. Thus, there is a pressing need for safer and more effective treatment strategies. In recent years, natural product-based therapies have garnered increasing attention due to their multi-target mechanisms and relatively low toxicity. This review comprehensively summarizes recent advances in the application of natural products for IDD treatment, with a focus on flavonoids (e.g., quercetin, hyperoside), glycosides (e.g., ginsenosides, notoginsenosides), terpenoids (e.g., aucubin, celastrol), phenolic compounds (e.g., curcumin, resveratrol), and alkaloids (e.g., berberine, evodiamine). These compounds exert their therapeutic effects by modulating critical signaling pathways, including Sirtuin-1 (SIRT1), Nuclear Factor-kappa B (NF-κB), Mitogen-Activated Protein Kinase (MAPK), Phosphoinositide 3-Kinase/Protein Kinase B (PI3K/Akt), and Nuclear Factor Erythroid 2–Related Factor 2 (Nrf2). Collectively, they exhibit potent anti-inflammatory, antioxidant, anti-apoptotic, anti-senescence, and regenerative properties. The insights presented herein provide a robust theoretical foundation to support future preclinical and clinical investigations, highlighting the considerable promise of natural products in IDD management.

## 1 Introduction

IDD describes a pathological process characterized by progressive structural and functional deterioration of intervertebral disc tissues due to multiple etiological factors. This degenerative condition predominantly affects middle-aged and elderly populations. With global population aging accelerating, IDD has emerged as one of the foremost causes of spine-related disability worldwide (Kos et al., 2019). The intervertebral disc, an integral component of the spinal structure, consists of the annulus fibrosus, nucleus pulposus (NP), and cartilaginous endplates (CEP), collectively essential for maintaining spinal stability and flexibility. Additionally, IDD is recognized as the primary pathological basis underlying disc herniation (Risbud and Shapiro, 2014). In a healthy state, intervertebral discs exhibit notable elasticity and effective shock-absorbing capabilities. Nevertheless, aging and external pathological factors progressively induce disc degeneration, typified by annular fissures, NP dehydration, and CEP calcification (Sar et al., 2016).

IDD contributes to various spinal disorders, including disc herniation, sciatica, and spinal stenosis. Present therapeutic strategies for IDD primarily aim at symptomatic relief and pain management (Le Maitre et al., 2015), employing both conservative and surgical interventions. Conservative approaches generally involve pharmacological therapies, physical rehabilitation, and lifestyle modifications. However, these methods typically provide only transient symptomatic relief and are largely ineffective in reversing the underlying degenerative processes (Yu et al., 2018). Surgical interventions, including discectomy and artificial disc replacement, can partially restore disc function, yet they carry substantial risks and potential postoperative complications (Cai et al., 2011). Moreover, the substantial economic burden associated with surgical treatment limits its accessibility. Consequently, the development of safer, more effective, and economically viable therapies remains a significant priority among clinicians and researchers. Recently, natural products have attracted considerable attention as potential therapeutic candidates for IDD due to their capability to modulate multiple biological targets involved in disc degeneration.

**FIGURE 1:**
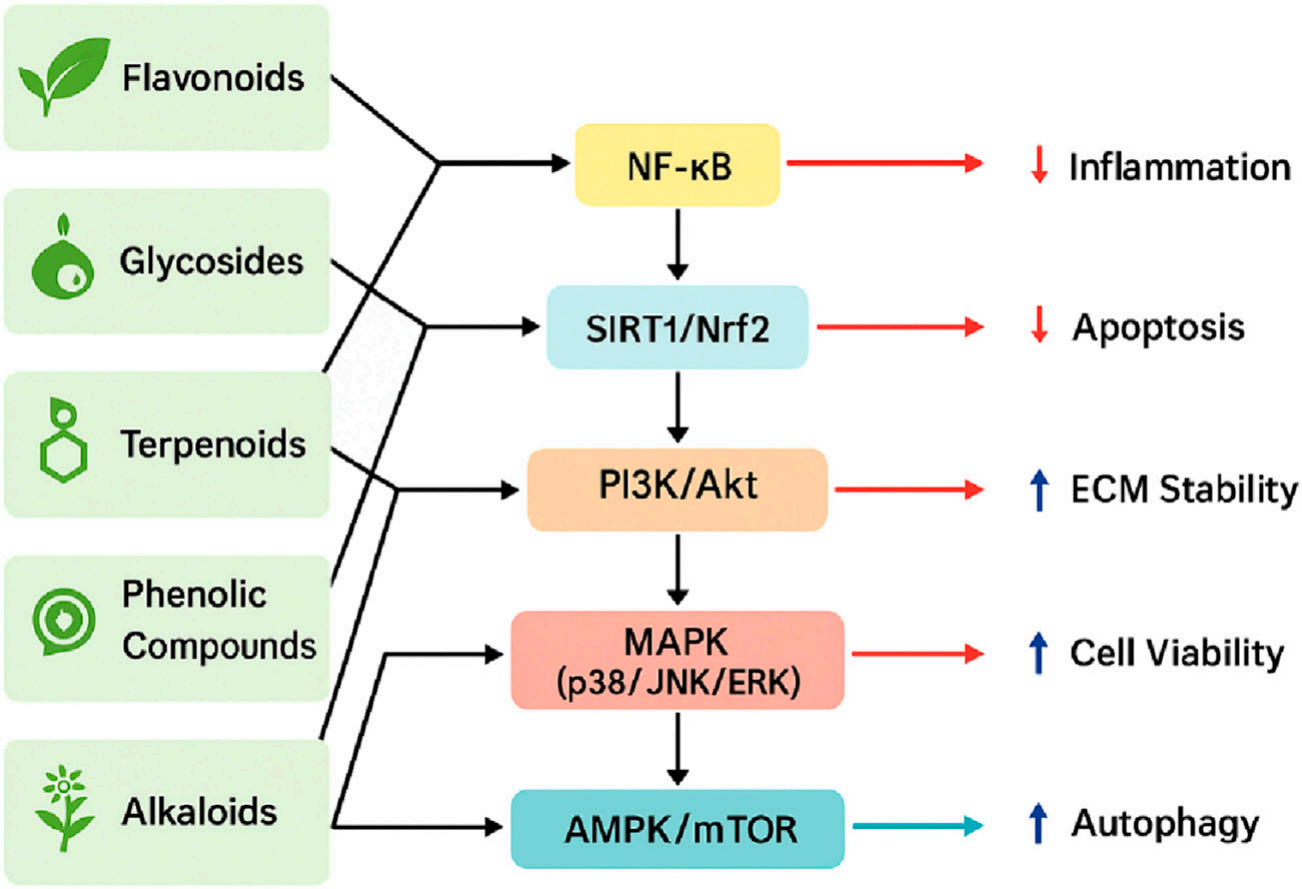
Schematic illustration of the major signaling pathways modulated by natural products in the treatment of IDD. Representative compound categories—including flavonoids, glycosides, terpenoids, phenolic compounds, and alkaloids—target key molecular pathways such as NF-κB, SIRT1/Nrf2, PI3K/Akt, MAPK (p38/JNK/ERK), and AMPK/mTOR. These pathways are involved in reducing inflammation and apoptosis, enhancing ECM stability and cell viability, and promoting autophagy, collectively contributing to the attenuation of IDD progression.

Natural products are chemical compounds derived from natural sources, including plants, animals, and microorganisms, characterized by a wide range of biological activities and pharmacological properties. Compared with traditional synthetic drugs, natural products offer significant advantages, such as lower toxicity and the capacity for multi-target interventions, making them increasingly attractive as therapeutic options for intervertebral disc degeneration (IDD) (Chen et al., 2021). These natural compounds have demonstrated substantial efficacy in reducing inflammation, alleviating oxidative stress, inhibiting apoptosis, and promoting cellular regeneration. Consequently, they may slow down or partially reverse the degenerative processes associated with IDD (Liu Z. et al., 2023; Mavrogonatou and Kletsas, 2024). Notably, flavonoids, glycosides, terpenoids, and phenolic compounds have demonstrated significant therapeutic promise in recent studies (Wan et al., 2025). This review comprehensively summarizes current advancements in utilizing natural products for IDD therapy, focusing specifically on representative compounds and elucidating their underlying mechanisms of action to guide future research and clinical development. These multi-targeted mechanisms and compound classifications are visually summarized in Figure 1.

Compared to previous reviews that primarily focused on inflammatory modulation or single-pathway mechanisms (Chen et al., 2021; Liu Z. et al., 2023), this study provides a systematic classification of natural compounds, integrates their molecular mechanisms across multiple pathways, and uniquely emphasizes structure-activity relationships and quantitative pharmacological data, offering a comprehensive perspective for future translational research.

A systematic literature search was conducted using PubMed, Web of Science, and Google Scholar, covering the period from January 2000 to May 2025. The following Boolean string was used (“intervertebral disc degeneration” OR “IVDD” OR “IDD”) AND (“natural products” OR “flavonoids” OR “alkaloids” OR “polyphenols” OR “glycosides” OR “terpenoids”). Articles were screened based on title and abstract, and inclusion criteria were: (1) studies reporting *in vitro* or *in vivo* effects of plant-derived metabolites on IDD; (2) studies involving known active ingredients with structural and mechanistic data; (3) peer-reviewed journal articles in English.

## 2 Flavonoids

**FIGURE 2:**
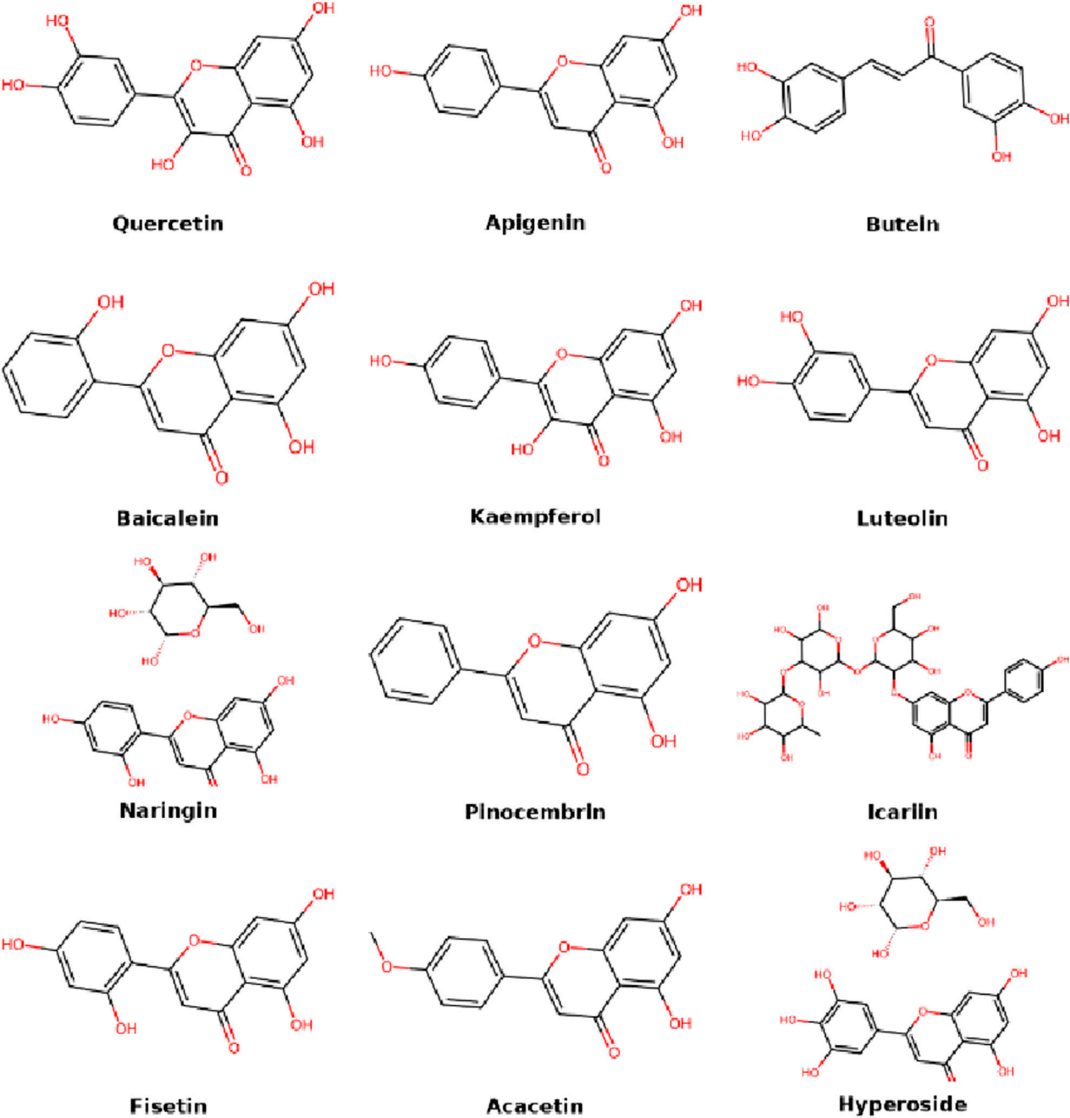
Molecular structures of flavonoid compounds.

Flavonoids are among the most widely investigated natural compounds in IDD treatment due to their potent antioxidant, anti-inflammatory, and matrix-preserving properties. Their polyphenolic structures allow them to scavenge free radicals, inhibit pro-inflammatory pathways, and modulate ECM metabolism in nucleus pulposus cells. It is worth noting that flavonoids are a subclass of phenolic compounds, sharing similar polyphenolic structures and biological functions such as antioxidation and anti-inflammation. However, flavonoids are often discussed separately due to their extensive subclass-specific research. This section discusses key flavonoids and their specific mechanisms of action, highlighting their therapeutic relevance to disc degeneration (Figure 2).

### 2.1 Hyperoside

Hyperoside is a natural flavonol glycoside derived from various plant genera, including *Hypericum*, *Filipendula*, and *Polygonum*. It is recognized for its notable anti-inflammatory, antioxidant, and anti-apoptotic activities (Xu et al., 2022). SIRT1, an NAD^+^-dependent deacetylase and key regulator of inflammation in inflammatory and immune responses (Han et al., 2021). Studies have indicated that SIRT1 activation stimulates the phosphoinositide 3-kinase (PI3K)/protein kinase B (Akt) signaling pathway, thereby suppressing interleukin (IL)-1β-induced apoptosis and inflammation in nucleus pulposus cells (NPCs), and regulating extracellular matrix (ECM) remodeling (Qi et al., 2020). Additionally, activation of endoplasmic reticulum (ER) stress contributes significantly to NPC apoptosis and ECM degradation, thereby playing a pivotal role in the pathogenesis of IDD (Yao et al., 2024). Recent evidence indicates that hyperoside significantly mitigates TNF-α-induced apoptosis in human NPCs by upregulating SIRT1 and Nrf2. Furthermore, hyperoside effectively reduces ECM degradation and apoptosis mediated by ER stress, highlighting its therapeutic potential in the treatment of IDD (Xie et al., 2022a).

### 2.2 Quercetin

Quercetin, a widely distributed flavonoid, exhibits strong antioxidant and anti-inflammatory effects (Li Y. et al., 2016). The p38 MAPK signaling pathway, an essential component of the MAPK family, significantly contributes to the pathological processes underlying intervertebral disc degeneration (IDD) (Zhang X. et al., 2023). Studies have shown that quercetin effectively protects NPCs from apoptosis by inhibiting p38 MAPK-mediated autophagy, thereby preventing ECM degeneration and significantly alleviating IDD symptoms in a rat tail puncture-induced IDD model (Zhang S. et al., 2021). Additionally, quercetin exerts protective effects on NPCs by suppressing apoptosis and ECM degradation through activation of the SIRT1-autophagy signaling pathway (Wang D. et al., 2020). Notably, quercetin is recognized as a senolytic agent capable of binding to the Kelch-like ECH-associated protein 1 (Keap1)–nuclear factor erythroid 2-related factor 2 (Nrf2) complex, subsequently inhibiting the NF-κB pathway. This action reduces the expression of senescence-associated secretory phenotype (SASP) factors in interleukin (IL)-1β-stimulated NPCs (Shao et al., 2021). Furthermore, a combination therapy involving dasatinib and quercetin has demonstrated efficacy in attenuating age-dependent IDD progression in mouse models (Novais et al., 2021). Quercetin also inhibits oxidative stress-induced senescence in mesenchymal stem cells derived from NPCs through regulation of the miR-34a/SIRT1 axis (Zhao WJ. et al., 2023). Quercetin (100 mg/kg/day) reduced IL-1β by 45% and increased SIRT1 2.1-fold. Network pharmacology studies have further identified quercetin as a critical bioactive component in traditional Chinese medicine formulas commonly employed for lumbar disc herniation treatment (Sun W. et al., 2022). These collective *in vitro* and *in vivo* findings underscore quercetin’s promising therapeutic potential for IDD management.

### 2.3 Apigenin

Apigenin, another flavonoid compound, possesses a broad spectrum of biological activities. Mammalian target of rapamycin (mTOR), a conserved serine/threonine kinase, has a significant role in various degenerative joint conditions, including IDD (Pal et al., 2015). The mTOR signaling pathway is essential for maintaining homeostasis within the intervertebral disc, and disruptions in autophagic flux are closely linked to NPC senescence and apoptosis during IDD progression (Chen et al., 2023). Apigenin enhances autophagy through the AMP-activated protein kinase (AMPK)/mTOR/transcription factor EB (TFEB) signaling cascade, effectively alleviating oxidative stress-induced senescence in NPCs. Moreover, apigenin suppresses the expression of tumor necrosis factor-alpha (TNF-α)-mediated pro-inflammatory cytokines, thereby mitigating disc degeneration in rat models of IDD (Ding and Li, 2020). These findings highlight apigenin as a potentially valuable natural therapeutic candidate for the prevention and treatment of IDD.

### 2.4 Butein

In addition to mechanical stress, diabetes and the accumulation of advanced glycation end-products (AGEs) significantly contribute to the progression of IDD (Li S. et al., 2024). Dysregulated glucose metabolism exacerbates IDD by affecting critical cellular processes such as senescence, apoptosis, inflammation, proliferation, and ECM degradation (Jung et al., 2023; Lintz et al., 2022). Butein, a chalcone-type flavonoid isolated from plants belonging to the Anacardiaceae, Asteraceae, and Fabaceae families (Padmavathi et al., 2017), exhibits diverse pharmacological properties, including antioxidant, anti-inflammatory, anti-angiogenic, anticancer, and antidiabetic activities (Song et al., 2016). Both *in vitro* and *in vivo* studies have revealed that butein activates SIRT1, leading to the suppression of p53 acetylation. This mechanism protects NPCs from apoptosis and senescence triggered by hyperglycemia. Specifically, butein treatment significantly alleviated IDD symptoms in diabetic rat models, as evidenced by increased expression of SIRT1 and decreased acetylation levels of p53 within nucleus pulposus tissues (Zh et al., 2019). These findings indicate butein as a promising candidate for IDD management, particularly in diabetic contexts.

### 2.5 Baicalein

Baicalein is a prominent flavonoid isolated predominantly from the roots of *Scutellaria baicalensis*, a traditional medicinal herb widely utilized in Chinese medicine (Tsou et al., 2016). It has been extensively studied for its potent hepatoprotective and anti-inflammatory properties across diverse disease models (Chen et al., 2014). Research has demonstrated that baicalein effectively inhibits the activation of key signaling pathways such as NF-κB and MAPK. It also reduces the overproduction of inflammatory cytokines, including prostaglandin E2 (PGE2), tumor necrosis factor-alpha (TNF-α), and IL-6 in IL-1β-stimulated NPCs. Baicalein (25–50 μM) reduced cyclooxygenase-2 (COX-2) and PGE2 by 52%–68%. Furthermore, baicalein has shown a dose-dependent capability to counteract ECM degradation, specifically reversing the loss of aggrecan and type II collagen (Col2) (Jin et al., 2019). Complementary *in vivo* studies employing rat models of needle puncture-induced IDD further validate baicalein’s therapeutic efficacy in mitigating disc degeneration (Jin et al., 2019). Additionally, baicalein suppresses TNF-α-induced apoptosis and catabolic activity in NPCs through activation of the PI3K/Akt signaling pathway (Liu Y. et al., 2023). Collectively, these studies underscore baicalein’s significant therapeutic potential as a natural product-based intervention for IDD.

### 2.6 Kaempferol

Kaempferol is a naturally occurring flavonoid found in tea, as well as many common vegetables and fruits, including legumes, broccoli, cabbage, gooseberries, grapes, kale, strawberries, tomatoes, citrus fruits, Brussels sprouts, apples, and grapefruits (Calderón-Montaño et al., 2011). Its anti-inflammatory properties allow it to be used in treating a range of acute and chronic inflammatory diseases, such as disc degeneration, colitis, postmenopausal bone loss, and acute lung injury (Ren et al., 2019). Bone marrow mesenchymal stem cells (BMSCs) are considered a promising autologous source for regenerating nucleus pulposus tissue. When co-cultured with NPCs, BMSCs can differentiate into NPC-like cells, enhancing their viability and matrix production (Cao et al., 2015). Kaempferol ameliorates IDD progression by suppressing osteogenic, adipogenic, and inflammatory responses induced by lipopolysaccharide (LPS) in BMSCs (Zhu et al., 2017). In an injectable kaempferol-loaded fibrin gel study (Gao et al., 2023), intradiscal injection in IDD rat models demonstrated favorable injectability, sustained release, and biocompatibility. The treatment reduced IDD-associated inflammation and regulated ECM synthesis and degradation. Network pharmacology studies suggest kaempferol may be a key active compound in traditional Chinese medicine for IDD (Wang X. et al., 2023; Liu H. et al., 2023). In IL-1β-induced *in vitro* IDD models, kaempferol inhibited phosphorylation of ERK1/2, downregulated matrix metalloproteinase (MMP)-3 and a disintegrin and metalloproteinase with thrombospondin motifs (ADAMTS)-4, while upregulating aggrecan and type II collagen expression. Kaempferol significantly restored cell viability and reduced both ROS accumulation and apoptosis in NPCs (Wang X. et al., 2023).

### 2.7 Luteolin

There is a close relationship between the intervertebral disc and the adjacent vertebral endplates, and Modic changes, defects, sclerosis, and calcification in these endplates are associated with disc degeneration (Zehra et al., 2019). Degeneration of the endplate may result in abnormal collagen-bone matrix remodeling, spatial reorganization, hypertrophy, and angiogenesis, which in turn promote IDD progression (Zehra et al., 2017). Luteolin, beyond its general antioxidant activity as a flavonoid, exhibits anti-inflammatory, cardiovascular, anticancer, and neuroprotective properties (Huan et al., 2023). It is found in various vegetables, botanical drugs, and fruits such as carrots, broccoli, cabbage, parsley, thyme, mint, basil, celery, artichokes, and apples (Aziz et al., 2020). A study on endplate chondrocytes found that luteolin significantly suppressed the expression of MMP13, p53, and p21 while promoting CDK2, CDK4, and Col2α1 expression. It alleviated chondrocyte senescence, as confirmed by cell cycle analysis, proliferation assays, and β-galactosidase staining (Long et al., 2025). Another study showed that Luteolin dose-dependently reduced NPC apoptosis and reversed TNF-α-induced senescence and inflammation through activating SIRT6 and inhibiting NF-κB (Xie et al., 2022b).

### 2.8 Naringin

Naringin is a flavonoid compound extracted from citrus fruits, known for its strong anti-inflammatory and antioxidant effects (Gan et al., 2023). In a study using degenerative human NPCs from patients with discogenic low back pain, naringin increased aggrecan, bone morphogenetic protein (BMP)-2, and SRY-box transcription factor 6 (Sox6) expression, while inhibiting TNF-α and MMP3 expression, promoting NPC proliferation and recovery from degeneration (Li N. et al., 2016). Naringin also enhanced autophagy by upregulating LC3 and Beclin-1, reducing oxidative stress-induced NPC apoptosis. Its anti-apoptotic effect was partially reversed by 3-methyladenine, suggesting autophagy is key to its protective action (Zhang et al., 2018). Naringin regulates autophagy *via* the AMPK pathway, either directly or by indirectly activating SIRT1 (Chen R. et al., 2022), maintaining ECM stability in terms of Col2, aggrecan, and MMP13. *In vivo* studies showed that naringin alleviated IDD in puncture-induced rat models. In studies on nucleus pulposus mesenchymal stem cells (NPMSCs), naringin at 100 μM for 24 h was non-cytotoxic and reduced H_2_O_2_-induced apoptosis *via* the PI3K/Akt pathway, also mitigating mitochondrial dysfunction, including increased ROS, decreased membrane potential, reduced ATP levels, and altered ultrastructure (Nan et al., 2020a). Naringin suppressed IL-1β-induced MMPs and inflammation in NPCs by downregulating the NF-κB pathway and p53 expression (Gao G. et al., 2019). It also inhibited annulus fibrosus cell apoptosis caused by cyclic stretch by suppressing NF-κB activation, and MRI assessments confirmed IDD alleviation in treated rats (Zhang YH. et al., 2022). Additionally, naringin protected endplate chondrocytes from apoptosis by promoting SIRT3-mediated mitophagy and suppressing NOD-like receptor family pyrin domain containing 3 (NLRP3) inflammasome activation (Wang J. et al., 2024), which is involved in the IDD pathological process (Chao-Yang et al., 2021). Naringin and its aglycone naringenin are also identified as effective anti-inflammatory agents for treating low back pain and sciatica (Devraj et al., 2019).

### 2.9 Pinocembrin

Pinocembrin is a major flavonoid compound isolated from various plants, including pine heartwood, eucalyptus, poplar, euphorbia, and *Boesenbergia rotunda* (Rasul et al., 2013). It exhibits antioxidant, anti-inflammatory, antimicrobial, neuroprotective, cardioprotective, and anticancer properties (Elbatreek et al., 2023). A significant study found that pinocembrin alleviated IDD progression in mice and protected the CEP from oxidative stress-induced degeneration and calcification (Wa et al., 2023). *In vitro*, it activated the Nrf2 pathway, inhibited parkin-mediated mitophagy, and reduced chondrocyte ferroptosis.

### 2.10 Icariin

Icariin is a flavonoid glycoside extracted from *Epimedium*, a traditional Chinese medicinal herb, and has gained attention due to its diverse pharmacological activities (Szabó et al., 2022). Widely used in traditional medicine and valued in modern pharmacology, icariin is a promising natural compound for biomedical and tissue engineering applications (Seyedi et al., 2023). It possesses multiple therapeutic effects on bone health, inflammation, cancer, immunity, cardiovascular and nervous system protection, and sexual function (Wang et al., 2021a; Song et al., 2020; Zeng et al., 2022; Si et al., 2024; Fang et al., 2024). Studies indicate that icariin protects NPCs and CEP cells and slows IDD progression by exerting anti-inflammatory, antioxidant, anti-apoptotic effects and promoting ECM synthesis. It inhibits IL-1β-induced MAPK and NF-κB pathways, reducing proinflammatory factors, degradative enzymes, and oxidative stress (Hua et al., 2018). Moreover, icariin activates the Nrf2/HO-1 pathway to promote mitophagy, inhibit ferroptosis, maintain mitochondrial function and redox balance, and enhance cell survival (Hua et al., 2020; Shao Y. et al., 2022). *In vivo*, it upregulates chemokines such as insulin-like growth factor (IGF)-1, transforming growth factor (TGF)-β, and stromal-derived factor (SDF)-1, promoting stem cell migration and tissue repair, mitigating CEP calcification and IDD pathology (Shao Y. et al., 2022; Zhang Z. et al., 2022).

### 2.11 Fisetin

Fisetin is a natural flavonoid found in many fruits and vegetables, such as strawberries, apples, persimmons, and cucumbers (Khan et al., 2013). Due to its antioxidant, anti-inflammatory, anticancer, anti-aging, and nephroprotective activities (Kashyap et al., 2019; Ren et al., 2021; Wang B. et al., 2024; Ding et al., 2022; Zhou C. et al., 2023), it shows potential in treating various chronic diseases. Fisetin protects both NPMSCs and NPCs by inhibiting oxidative stress and apoptosis, while maintaining ECM integrity (Zhou Q. et al., 2023). Oxidative stress is a major driver of IDD, and fisetin, acting through the Nrf2/HO-1 pathway, inhibits oxidative stress-induced ferroptosis, reduces cell death, and maintains ECM homeostasis (Li C. et al., 2024). Both *in vitro* and *in vivo* studies confirm its effectiveness in protecting disc cells and alleviating disc degeneration in rats (Zhou Q. et al., 2023; Li C. et al., 2024).

### 2.12 Acacetin

Acacetin is a monomethoxy flavonoid mainly found in *Robinia pseudoacacia* and various botanical drugs (Singh et al., 2020). It has shown broad therapeutic potential due to its anti-inflammatory, antimicrobial, antioxidant, anticancer, anti-obesity, and cardiovascular protective properties (Wu et al., 2022; Zhang G. et al., 2023; Bu et al., 2024; Mu et al., 2022; Liou et al., 2022; Li et al., 2020). Acacetin effectively mitigates NPC degeneration in both *in vitro* and *in vivo* models. *In vitro*, it activates the Nrf2 pathway, upregulates antioxidant proteins such as HO-1, NAD(P)H:quinone oxidoreductase 1 (NQO1), and superoxide dismutase (SOD), inhibits ROS production, and reduces COX-2 and iNOS-mediated inflammation. It also prevents aggrecan and Col2 degradation (Wang et al., 2020b; Pan C. et al., 2023). Additionally, acacetin inhibits the phosphorylation of p38, c-Jun N-terminal kinase (JNK), and ERK1/2, thereby slowing NPC degeneration. *In vivo* studies using MRI and histopathology confirmed that acacetin significantly ameliorates IDD in puncture-induced rat models (Wang et al., 2020b).

## 3 Glycosides

**FIGURE 3:**
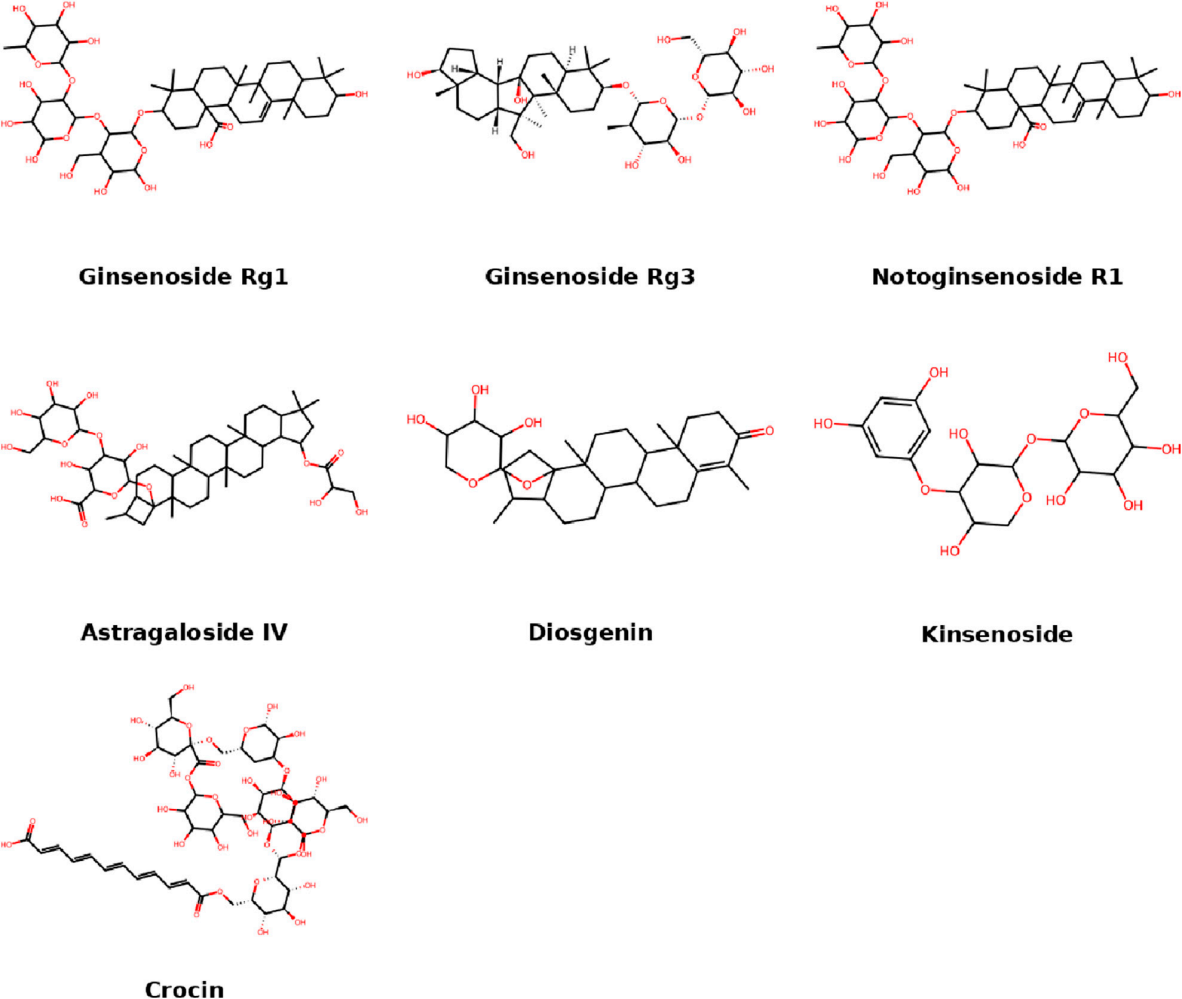
Molecular structures of glycosides compounds.

Glycosides, especially saponins, are active components in many traditional herbal medicines. In IDD models, glycosides exert anti-inflammatory, antioxidative, and anti-apoptotic effects, often through PI3K/Akt or JAK/STAT signaling. Their glycosidic linkages enhance solubility and bioavailability, contributing to their therapeutic potential. This section summarizes representative glycosides and their regulatory functions in IDD (Figure 3).

### 3.1 Ginsenosides

Ginseng is one of the most widely used herbal nutraceuticals in the world and has a long history of use in traditional Chinese medicine (Shi et al., 2019). Modern pharmacological studies have shown that ginseng has multiple biological effects, including anticancer, antioxidant, and anti-inflammatory activities (Metwaly et al., 2019). Ginsenosides are the main active components of ginseng and are triterpenoid saponins, with more than 180 types identified to date (Yu et al., 2019). Based on the structure of the glycoside moiety, Rg1 is classified as a protopanaxatriol-type dammarane ginsenoside (Kim et al., 2015). Ginsenoside Rg1 has become a major research focus for IDD treatment. It can regulate disc homeostasis and water content, inhibit apoptosis, inflammation, and ECM degradation, thereby delaying the progression of IDD. Rg1 improves NPC proliferation, reduces apoptosis, and enhances aggrecan and type II collagen (Col2α1) expression by inhibiting the Wnt/β-catenin signaling pathway (Yu et al., 2020). It also suppresses the activation of the Yes-associated protein (YAP)-1/transcriptional coactivator with PDZ-binding motif (TAZ) pathway, thereby preventing IDD progression. Rg1 significantly increases the mechanical and thermal threshold in IDD rats and alleviates histological changes (Yang YH. et al., 2022). Recent studies found that Rg1 ameliorates IDD progression in rats by inhibiting the activation of the NF-κB pathway. In IL-1β-induced NPCs, Rg1 promotes proliferation, inhibits apoptosis, and suppresses the expression of IL-6, TNF-α, aggrecan, collagen II, p-p65/p65, and inhibitor of kappa B kinase (IκK) in a dose-dependent manner (Yu et al., 2024). Rg1 (10–50 μM) decreased TNF-α and IL-6 by 60%, and upregulated aggrecan expression by >2-fold. In addition, ginsenoside Rg3, a protopanaxadiol-type saponin, has also been shown to regulate IDD. Treatment with Rg3 reversed IL-1β-induced apoptosis in NPCs and significantly reduced the expression of MMP2, MMP3, ADAMTS-4, and ADAMTS-5 *via* inactivation of the p38 MAPK pathway. Compared to Rg1, Rg3 not only alleviates NPC degeneration but also restores annulus fibrosus alignment and preserves more proteoglycan-rich matrix (Chen J. et al., 2024).

### 3.2 Notoginsenoside

The root of *Panax notoginseng* has been used as a traditional herb for thousands of years, mainly for hemostasis and promoting blood circulation, and it holds a landmark status in traditional Chinese medicine (Zhu and Wan, 2023). Its major pharmacological effects are attributed to notoginsenosides, a group of dammarane-type tetracyclic triterpenes with potent bioactivity (Liu et al., 2020a). Notoginsenosides exhibit a broad range of activities including cardiovascular protection, neuroprotection, antidiabetic effects, hepatoprotection, gastrointestinal protection, pulmonary protection, bone metabolism regulation, renal protection, and anticancer effects (Liu et al., 2020b; Guo et al., 2019). Notoginsenoside R1 (NGR1), a member of the protopanaxatriol group, is the major component of notoginsenosides, with significantly higher content in roots and rhizomes than in other plant parts (Zhu T. et al., 2021). NGR1 enhances alkaline phosphatase activity and mineralized nodule formation in bone marrow mesenchymal stem cells (BMSCs), and increases estrogen receptor-α expression, thereby regulating the GSK-3β/β-catenin pathway to promote BMSC proliferation, migration, and osteogenic differentiation (Lu et al., 2024). In studies using IDD rat models and NPCs, NGR1 inactivated the NF-κB/NLRP3 pathway, improved NPC function, and inhibited pyroptosis, while promoting ECM synthesis and reducing proinflammatory cytokine mRNA expression both *in vitro* and *in vivo* (Tang et al., 2021).

### 3.3 Astragaloside IV

Astragalosides I, II, and IV are the major saponins found in *Astragalus membranaceus*, with astragaloside IV (AS-IV) being the most biologically active (Zhang et al., 2020). AS-IV is one of the main active components extracted from *Astragalus* and is considered a marker compound for quality evaluation of traditional Chinese medicines. It has demonstrated anti-inflammatory, antioxidant, neuroprotective, antifibrotic, and antitumor effects (Liang et al., 2023). In NPCs, miR-223 promotes inflammation and cell injury *via* the JAK2/STAT1 pathway, and the combined use of AS-IV and tanshinone IIA may protect NPCs by downregulating miR-223 and suppressing JAK2 and STAT1 expression (Du et al., 2021). Other *Astragalus* derivatives, such as cycloastragenol and AS-IV, have been shown to extend the proliferative capacity and lifespan of NPCs (Hong et al., 2021). These compounds upregulate telomerase expression and improve telomere attrition under high glucose conditions, while enhancing proliferation and morphology of NPCs. *In vitro* and *in vivo* studies of IDD have demonstrated that AS-IV reduces IL-1β-induced inflammation, apoptosis, and ECM degradation, and protects against IDD progression in needle-puncture rat models (Tian et al., 2022). AS-IV inhibits IκB-α phosphorylation and NF-κB p65 nuclear translocation, indicating suppression of the NF-κB pathway. It also upregulates Col2, aggrecan, and Bcl-2 while downregulating Bax and cleaved caspase-3 expression and activating the PI3K/Akt pathway (Zhang L. et al., 2023). Furthermore, AS-IV maintains disc height and volume in lumbar instability mouse models, improves matrix metabolism, and restores Col2α1, ADAMTS-5, aggrecan, and MMP-13 expression in degenerated discs. It also suppresses EGFR, p38 MAPK, and caspase-3 expression in annulus fibrosus tissue during IDD progression, possibly *via* inhibition of the EGFR/MAPK pathway (Chen D. et al., 2024).

### 3.4 Dioscin

Dioscin is a natural steroidal saponin with bioactivity, extracted from several medicinal botanical drugs (Bandopadhyay et al., 2022). Pharmacological research has demonstrated its anti-inflammatory, anti-apoptotic, and antioxidant effects in various diseases (Tao et al., 2018). In IDD, dioscin inhibits the IL-1β-induced overexpression of MMP1, MMP3, MMP13, and ADAMTS-5, while promoting Col2 and aggrecan synthesis, thereby maintaining ECM homeostasis in cartilage. These effects are associated with inhibition of the MAPK and NF-κB signaling pathways (Ding et al., 2023). Toll-like receptor 4 (TLR4) is overexpressed in cartilage during osteoarthritis and plays an important role in cartilage degradation (Gómez et al., 2015). Similarly, degenerated NPCs show increased TLR4 expression and respond to LPS-induced TLR4 activation by enhancing proinflammatory cytokine release and reducing ECM content in discs (Rajan et al., 2013). Studies on the potential effects of dioscin in IL-1β-treated NPCs indicate that it suppresses the TLR4/NF-κB pathway to reduce catabolic activity and levels of IL-6 and TNF-α (Wang L. et al., 2020).

### 3.5 Kinsenoside

Kinsenoside is a glycoside compound extracted from *Anoectochilus roxburghii* and is considered its primary bioactive constituent (Qi et al., 2018). *Anoectochilus* is a member of the Orchidaceae family and is widely distributed in tropical and subtropical Asia (Song W. et al., 2021). Kinsenoside possesses hepatoprotective, hypoglycemic, hypolipidemic, anti-inflammatory, vasoprotective, and anti-osteoporotic properties (Han et al., 2016; Zhang et al., 2007; Liu et al., 2013; Xiang et al., 2016). Its biological effects are associated with pathways such as ERK, MAPK, NF-κB, and vascular endothelial growth factor (VEGF) signaling (Luo et al., 2018). In both *in vivo* and *in vitro* studies, kinsenoside treatment alleviated T2-weighted signal loss and disc height reduction in IDD rat models, kinsenoside (50 mg/kg) improved disc height index by 22.3%, improved matrix loss and other pathological features, and delayed IDD progression. Kinsenoside activated the AKT–ERK1/2–Nrf2 pathway in NPCs, and in a Nrf2-dependent manner, rescued NPC viability under oxidative stress and protected against apoptosis, senescence, and mitochondrial dysfunction (Wang Y. et al., 2019).

### 3.6 Crocin

Crocin is a glycosylated carotenoid compound extracted from *Crocus sativus L* (saffron) and is the main water-soluble carotenoid responsible for its yellow color (Boozari and Hosseinzadeh, 2022). Crocin has demonstrated antioxidant, anti-inflammatory, neuroprotective, anti-retinopathy, anticancer, and antidepressant properties (Heydari et al., 2023; Hashemzaei et al., 2020; Pourmousavi et al., 2024; Tao et al., 2023). Studies have shown that crocin can inhibit inflammation and catabolic processes associated with IDD (Li et al., 2015). *In vitro*, crocin significantly suppresses the LPS-induced overexpression of MMP-1, MMP-3, MMP-13, ADAMTS-4, ADAMTS-5, and proinflammatory cytokines including IL-1β, TNF-α, IL-6, iNOS, and TLR-2. Crocin (25–100 μM) reduced MMP-13 mRNA by 65%, cytokine levels by >50%. It also inhibits JNK phosphorylation in the MAPK pathway and partially prevents the reduction of chondroitin sulfate and Col2. *Ex vivo* experiments indicate that crocin protects ECM components in the disc and delays IDD progression (Li et al., 2015).

## 4 Terpenoids

**FIGURE 4:**
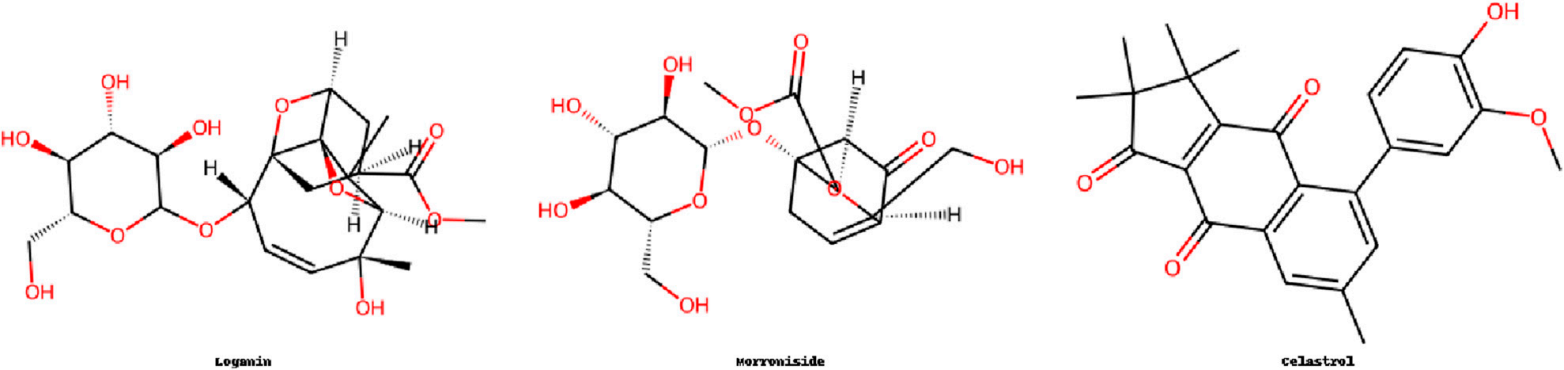
Molecular structures of terpenoids compounds.

Terpenoids, derived from isoprene units, exhibit diverse bioactivities and have shown promise in IDD therapy. Their mechanisms include the inhibition of inflammatory signaling pathways, protection of ECM components, and modulation of cellular stress responses. Glycosides, especially saponins, sometimes overlap in biological functions with terpenoids, particularly in modulating PI3K/Akt and NF-κB pathways. This overlap indicates possible structural synergy or shared biosynthetic origins. This section highlights key terpenoid compounds and their functions in delaying disc degeneration (Figure 4).

### 4.1 Aucubin

Aucubin is an iridoid glycoside compound widely found in traditional medicinal botanical drugs such as *Eucommia ulmoides Oliv (*Eucommiaceae*)*, *Aucuba japonica*, and *Plantago asiatica* (Bridi et al., 2023). It exhibits anti-inflammatory, antioxidant, anxiolytic and antidepressant, antidiabetic, antifibrotic, antimicrobial, anticancer, antihypertensive, gastroprotective, cardioprotective, and retinoprotective properties (Kartini et al., 2023; Wang et al., 2020d; Shao M. et al., 2022; Yang P. et al., 2022; Feng et al., 2023). Due to its abundant natural sources, high safety, and multiple biological benefits, aucubin holds great potential for applications in health supplements and pharmaceuticals (Zeng et al., 2020). In recent years, microRNAs (miRNAs) have been actively studied in the context of IDD and are considered to play important roles in its pathogenesis through various pathways (Wang et al., 2022). Aucubin inhibits ECM degradation in IL-1β- or TNF-α-stimulated NPCs by downregulating miR-140 expression and modulating its downstream target, cAMP response element-binding protein 1 (CREB1) (Ya et al., 2019). In both *in vivo* and *in vitro* experiments using a lumbar instability mouse model, aucubin was found to increase the expression of Col2α1 and aggrecan while reducing MMP-13, p-p65, NLRP3, and caspase-1 expression. It suppressed NF-κB–NLRP3 inflammasome activation in chondrocytes and mitigated ECM degradation in cartilage endplate (CEP) cells, thereby alleviating CEP degeneration (Zou et al., 2023). Aucubin (100 mg/kg/day) increased Col2α1 and aggrecan expression by 2.5-fold, and decreased MMP-13 by 60%. Additionally, aucubin exerts protective effects against IDD by modulating the NF-κB and Wnt signaling pathways, inhibiting cellular senescence, and reducing inflammatory cytokine levels (Li L. et al., 2023).

### 4.2 Morroniside

Morroniside is an iridoid glycoside extracted from *Cornus officinalis*, a traditional herb that has been used as food and medicine in China, Korea, and Japan for over 2,000 years (Shi P. et al., 2024). It possesses neuroprotective, osteoprotective, cardioprotective, nephroprotective, and hepatoprotective properties (Yu et al., 2021; Li et al., 2023b; Li W. et al., 2023; Zhang C. et al., 2024), and has demonstrated potential in the prevention and treatment of focal cerebral ischemia, spinal cord injury, Alzheimer’s disease, osteoporosis, osteoarthritis, acute myocardial infarction, and diabetes (Jiang H. et al., 2024; Xiao et al., 2023; Qian et al., 2024; Duan et al., 2021; Chen Y. et al., 2024). Recent studies have shown that morroniside significantly ameliorates the progression of IDD. In both *in vitro* and *in vivo* studies, morroniside suppressed ROS-induced aberrant activation of the Hippo signaling pathway in NPCs and reduced the expression of senescence markers including senescence-associated β-galactosidase, p53, and p21 (Zhou et al., 2022; Ge et al., 2024). Its mechanisms include inhibition of the phosphorylation of MST1/2 and LATS1/2 within the Hippo pathway, thereby reversing YAP/TAZ suppression, alleviating NPC senescence, and mitigating IDD progression by regulating ECM metabolism and preserving tissue structural integrity (Zhou et al., 2022). Moreover, morroniside reduced NPC pyroptosis by activating the Nrf2/Keap1 pathway, further supporting its therapeutic potential in IDD (Ge et al., 2024). These findings suggest that morroniside may serve as a novel therapeutic agent for IDD by targeting multiple mechanisms, particularly the ROS–Hippo–p53 signaling axis.

### 4.3 Celastrol

Celastrol is a pentacyclic triterpenoid compound extracted from the traditional Chinese medicine *Tripterygium wilfordii Hook. f. (*Celastraceae*)*, and it exhibits a wide range of pharmacological effects (Wang et al., 2023b). It has shown potent anticancer, antitumor, anti-obesity, and antidiabetic properties (Song et al., 2023; Xu H. et al., 2023; Xu et al., 2021; Gu et al., 2023), and has demonstrated unique therapeutic potential for acute and chronic inflammation, brain injury, vascular disorders, immune diseases, renal disorders, skeletal diseases, and cardiac conditions (Li Z. et al., 2022; Li M. et al., 2022; Zhang C. et al., 2021; Shirai et al., 2023; Pan M. et al., 2023). Celastrol effectively suppresses inflammation and oxidative stress by inhibiting the NF-κB signaling pathway, reducing IL-1β-induced expression of matrix-degrading enzymes (such as MMP-3, MMP-9, MMP-13, and ADAMTS-4, -5), oxidative stress markers (COX-2, iNOS), and pro-inflammatory cytokines (IL-6, TNF-α) in NPCs (Chen et al., 2017). Celastrol (0.25–1.0 μM) reduced MMP-13 and ADAMTS-5 by over 70%. Moreover, *in vivo* studies have shown that celastrol improves disc structure and significantly enhances T2-weighted MRI signals in a puncture-induced rat model of IDD (Chen et al., 2017).

## 5 Phenolic

**FIGURE 5:**
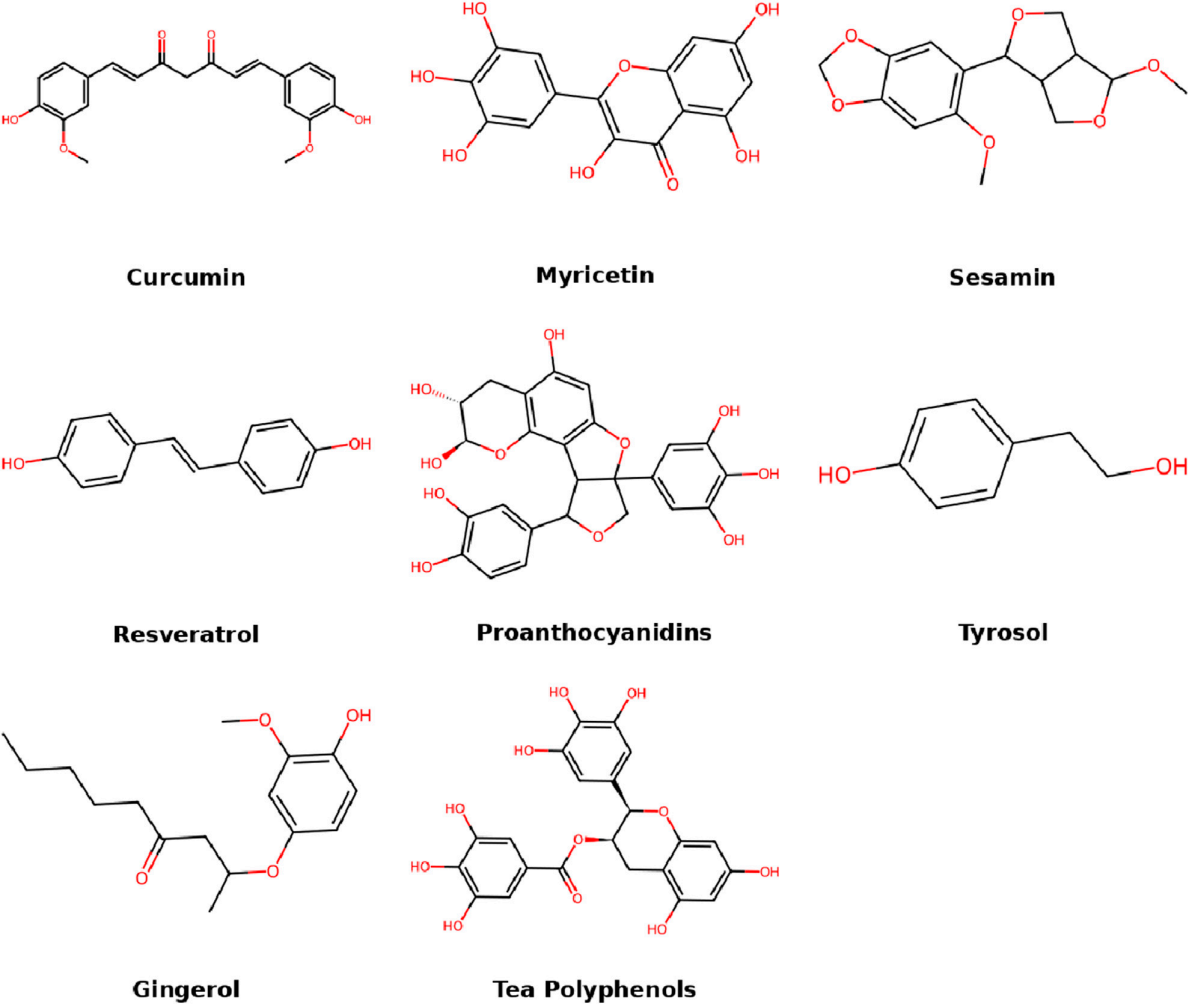
Molecular structures of phenolic compounds.

Some terpenoids exhibit similar anti-inflammatory mechanisms to glycosides and phenolics, suggesting cross-class pharmacological activity in IDD management. Phenolic compounds are natural antioxidants widely found in plants. In IDD, they modulate oxidative stress, inhibit inflammatory mediators, and promote ECM synthesis. Their structural diversity, such as hydroxyl group positioning and conjugation, contributes to their differential biological effects. Phenolic compounds as a broad category include flavonoids as a subclass. Their shared hydroxyl-rich structures contribute to their roles in redox regulation and inflammation suppression. This section reviews major phenolic agents involved in IDD management (Figure 5).

### 5.1 Curcumin

*Curcuma longa* (Zingiberaceae) is widely used in India, China, and Southeast Asia as an aromatic stimulant, food preservative, and coloring agent. It is now also cultivated in other regions, including Southeast Asia, China, and Latin America (Kotha and Luthria, 2019). Curcumin, a polyphenolic compound extracted from turmeric, possesses a wide range of pharmacological properties, including anti-aging, anti-inflammatory, antioxidant, anticancer, antidiabetic, antibacterial, antiviral, antifibrotic, immunomodulatory, and antifungal activities (Zia et al., 2021; Abd et al., 2021; Zhao C. et al., 2023; Nanavati et al., 2022). In recent years, curcumin nanoformulations have attracted increasing attention—such as nanofibers, lipid-based nanostructured carriers, solid self-nanoemulsifying drug delivery systems, and nanohydrogels—which offer enhanced bioavailability compared to free curcumin (Ataei et al., 2023; Ansari et al., 2023; Araya-Sib et al., 2021). In IDD rat models, curcumin intervention significantly reduced the expression of NF-κB p65 and TNF-α in lumbar disc tissues. MRI and ultrastructural analyses also revealed marked improvements in disc degeneration in the curcumin-treated group (Ma et al., 2015). Further studies found that curcumin protects against IDD progression by decreasing levels of IL-1β, IL-6, iNOS, COX-2, TGF-β1/2, and MMP-9, while promoting the expression of brain-derived neurotrophic factor (BDNF) (Hu et al., 2017). Curcumin (50 mg/kg) reduced NF-κB and TNF-α by 45%–60%, BDNF ↑2.3-fold. Curcumin regulates the expression of senescence-associated secretory phenotype (SASP) factors and enhances ECM synthesis *via* the Nrf2 and NF-κB pathways in degenerated discs (Cherif et al., 2019). In addition, curcumin upregulates autophagy, inhibits apoptosis, and mitigates phenotype loss in endplate chondrocytes under high-tension mechanical loading, thereby alleviating mechanical imbalance-induced IDD (Xiao et al., 2020). Novel polylactic acid nanoparticles loaded with curcumin—produced using solvent evaporation and water-in-oil emulsion methods—form bioactive hydrogels that overcome curcumin’s hydrophobicity and inhibit TNF-α production, immune cell activation, and inflammation, offering a promising future strategy for IDD therapy (Zamboni et al., 2022).

### 5.2 Myricetin

Myricetin is both a flavonoid and a phenolic compound. Its structure contains multiple hydroxyl groups, giving it strong antioxidant capacity typical of polyphenols (Jomová et al., 2019). Myricetin is widely distributed in fruits, vegetables, tea, berries, and red wine (Song X. et al., 2021). It exhibits antioxidant, antidiabetic, anticancer, anti-inflammatory, antiepileptic, anti-amyloidogenic, and cardiovascular protective properties (Kumar et al., 2023; Pluta et al., 2021; Wang L. et al., 2019; Wang et al., 2023c; Rahmani et al., 2023; Sharma et al., 2023; Ma et al., 2022). Studies have shown that myricetin inhibits IL-1β-induced inflammatory responses, reducing the production of proinflammatory mediators such as iNOS, COX-2, TNF-α, and IL-6, while regulating ECM component expression—reversing downregulation of aggrecan and type II collagen and suppressing the upregulation of MMP-13 and ADAMTS-5 (Xie et al., 2023). Myricetin (25–50 μM) reduced IL-6/iNOS 58%–70%. Additionally, myricetin activates the Nrf2/HO-1 signaling pathway and blocks NF-κB activation, protecting NPCs from oxidative stress and inflammation (Mao and Fan, 2024). In both *in vitro* and *in vivo* models, myricetin effectively alleviated apoptosis, mitochondrial dysfunction, and senescence induced by H_2_O_2_ or IL-1β (Xie et al., 2023; Mao and Fan, 2024). These findings highlight myricetin’s significant biological activity in preventing and treating IDD, particularly through the Nrf2/HO-1 pathway.

### 5.3 Sesamin

Sesamin is a lipophilic lignan classified as a polyphenol, derived from sesame seeds and oil. It exhibits diverse pharmacological activities including immunomodulation, anti-inflammation, antioxidant, and neuroprotection (Majdalawieh et al., 2022; Dalibalta et al., 2020; Ghaderi et al., 2023). It is commonly used as a dietary supplement to improve blood pressure and lipid levels, mainly by modulating key steps in fatty acid and cholesterol metabolism (Sun Y. et al., 2022; Majdalawieh et al., 2020). In studies of disc degeneration, sesamin protects intervertebral discs from inflammation and ECM damage by inhibiting JNK phosphorylation and MAPK pathway activation, thereby reducing LPS-induced expression of inflammatory cytokines and catabolic enzymes (Li K. et al., 2016). *In vivo*, sesamin attenuates injury-induced IDD, as demonstrated by preserved MRI signals, suppressed expression of catabolic enzymes, maintained ECM content, and reduced histological degeneration, indicating its potential as an early therapeutic agent for IDD (Li and Lv, 2020). Moreover, sesamin inhibits apoptosis, reverses CASP3, BAX, and BCL2 expression, delays ECM degradation, and promotes cell proliferation, showing protective and therapeutic effects against lumbar disc degeneration (Guo et al., 2023). Recent studies have found that sesamin enhances cell viability and reduces apoptosis by upregulating BECN2 and downregulating autophagy-related genes (ATG14, VPS34, GASP1) and inflammasome proteins (NLRP3, NLRC4, NLRP1, AIM2), alleviating LPS-induced chondrocyte degeneration. These findings support BECN2 as a potential target for IDD therapy (Zhang B. et al., 2024).

### 5.4 Tea polyphenols

Tea polyphenols (TPs) possess anti-inflammatory, antioxidant, immunomodulatory, and antitumor properties (Hong et al., 2022; Wang et al., 2021b; Dai et al., 2023). Major active components such as epigallocatechin gallate (EGCG) and epicatechin gallate (ECG) have shown promise in treating immune-related disorders and suppressing tumor metastasis (Bag and Bag, 2020). In food processing, TPs interact with proteins, polysaccharides, and lipids, affecting their functional properties (Rana et al., 2022). Further research into their immunomodulatory, antitumor mechanisms, and nutritional interactions will provide scientific evidence for health promotion and disease prevention. Studies have confirmed that TPs activate the Keap1/Nrf2/ARE pathway, enhance the expression of matrix-related genes, and reduce degeneration-associated factors, thereby protecting NPCs from oxidative stress-induced degeneration and effectively delaying IDD in both *in vitro* and *in vivo* models (Song D. et al., 2021).

### 5.5 Resveratrol

Resveratrol exhibits multiple mechanisms of action in delaying or treating IDD, primarily by modulating the SIRT1 pathway, inhibiting ECM degradation and inflammation, and delaying cellular senescence. In degenerated human NPCs, resveratrol significantly upregulates SIRT1, Col2α1, and aggrecan expression while downregulating MMP-1 (Wu et al., 2015). In rabbit IDD models, intradiscal injection of resveratrol improved T2-weighted MRI signals, increased aggrecan expression, and reduced MMP-13 mRNA levels (Kwon, 2013). Mechanistically, resveratrol activates SIRT1, suppresses NF-κB signaling, and in 1,25(OH)_2_D-deficient mice, reduces TNF-α and IL-1β levels *via* SIRT1-mediated p65 deacetylation (Wang P. et al., 2023). Furthermore, resveratrol regulates NPC autophagy *via* the Nampt/NAD^+^/SIRT1 pathway, restoring LC3 II/I and Beclin-1 expression and delaying degeneration (Shi et al., 2022). Resveratrol (20 μM) increased LC3-II by >2-fold, reduced apoptosis 40%. For delivery, thermosensitive PLGA–PEG–PLGA hydrogels offer controlled release, and co-delivery with tannic acid significantly suppresses local inflammation and promotes ECM regeneration (Liu et al., 2025). Targeted delivery is further improved using CDH2 antibody-loaded nanobubbles with ultrasound, enhancing resveratrol localization and release in NPCs, effectively slowing degeneration (Shen et al., 2018). Resveratrol also exhibits anti-apoptotic, anti-aging, and antioxidant effects, inhibits p21 and p16 expression, promotes cell proliferation, and reduces apoptosis, supporting its potential as a therapeutic agent for IDD (Liu et al., 2022; Guo et al., 2017).

### 5.6 Proanthocyanidins (PACs)

Proanthocyanidins (PACs) effectively delay the progression of IDD through multi-target mechanisms, exhibiting significant anti-apoptotic, anti-aging, antioxidant, and anti-inflammatory properties (Xu J. et al., 2023). Studies have shown that PACs activate the PI3K/Akt pathway to upregulate Bcl-2 expression and suppress Bax and cleaved caspase-3, thereby reducing IL-1β-induced apoptosis in human NPCs. PACs also inhibit the p53/p21/p16 signaling pathway, thereby reducing cellular senescence (Chen HW. et al., 2022). With respect to mitochondrial homeostasis, PACs help maintain membrane potential by activating the SIRT3/FOXO3 axis, inhibit Drp1-mediated mitochondrial fission, and promote the expression of OPA1 and MFN2, significantly reducing caspase-3 activity (Hua et al., 2024). In terms of inflammation regulation, PACs block the binding of LPS to TLR4/MD-2, suppress the NF-κB pathway, and reduce the expression of various inflammatory mediators and matrix-degrading enzymes, while promoting the synthesis of type II collagen and aggrecan (Shang et al., 2020). Despite the current evidence being primarily derived from cellular and animal models, PACs show promising potential as therapeutic candidates for IDD, warranting the development of targeted delivery systems and further evaluation for clinical translation.

### 5.7 Tyrosol

Tyrosol is a natural phenolic compound with the chemical structure 4-(2-hydroxyethyl)phenol, widely found in marine and terrestrial fungi (e.g., *Penicillium* species) and plant endophytes (Ferreira et al., 2025). It exhibits a diverse range of activities, including inhibition of pathogenic virulence factor expression, antitumor and anti-inflammatory effects, as well as regulation of intestinal metabolism, showing application potential in antimicrobial, anticancer, and metabolic disease contexts (Chang et al., 2019; Yu et al., 2023; Pal et al., 2023). Tyrosol significantly inhibits IL-1β-induced apoptosis and inflammation in human NPCs by upregulating SIRT1 and activating the PI3K/Akt pathway, reducing caspase activity and the levels of TNF-α, IL-6, nitric oxide (NO), and prostaglandin E2 (PGE2). It also suppresses MMP expression while promoting the synthesis of type II collagen, SOX-9, and aggrecan. These effects are mediated by SIRT1-dependent modulation of the NF-κB/FOXO3 pathway, suggesting the potential of tyrosol as a therapeutic agent for delaying IDD progression (Qi et al., 2020).

### 5.8 Gingerol

Gingerols are phenylpropanoid phenolic compounds found in the rhizome of ginger, characterized by an o-methoxyphenol group and an unsaturated C11–C15 carbon chain. They are subclassified into 6-, 8-, and 10-gingerols based on side-chain length (Martin et al., 2017). Gingerols exhibit multiple biological activities, including anti-inflammatory, anticancer, antidiabetic, and antioxidant effects (Nimmakayala et al., 2016; Basith et al., 2016; Orhan et al., 2007). Gingerol derivatives such as D-6-G and 6-gingerol (6-GIN) have shown effectiveness in delaying IDD through multiple mechanisms. D-6-G significantly suppresses NLRP3 inflammasome activation and GSDMD-mediated pyroptosis, reduces IL-1β and IL-18 secretion, and upregulates IL-10. It also activates the Nrf2/HO-1 pathway to scavenge reactive oxygen species (ROS) and preserve mitochondrial function (Xi et al., 2024). 6-GIN simultaneously activates the PI3K/Akt pathway, enhances type II collagen and aggrecan expression, and inhibits MMP-13 along with apoptosis-related signaling, and enhances autophagy to clear damaged mitochondria. Its protective effects on ECM depend on PI3K/Akt signaling, highlighting its anti-inflammatory, antioxidant, and matrix-repairing potential in IDD (Nan et al., 2020b).

## 6 Alkaloids

**FIGURE 6:**
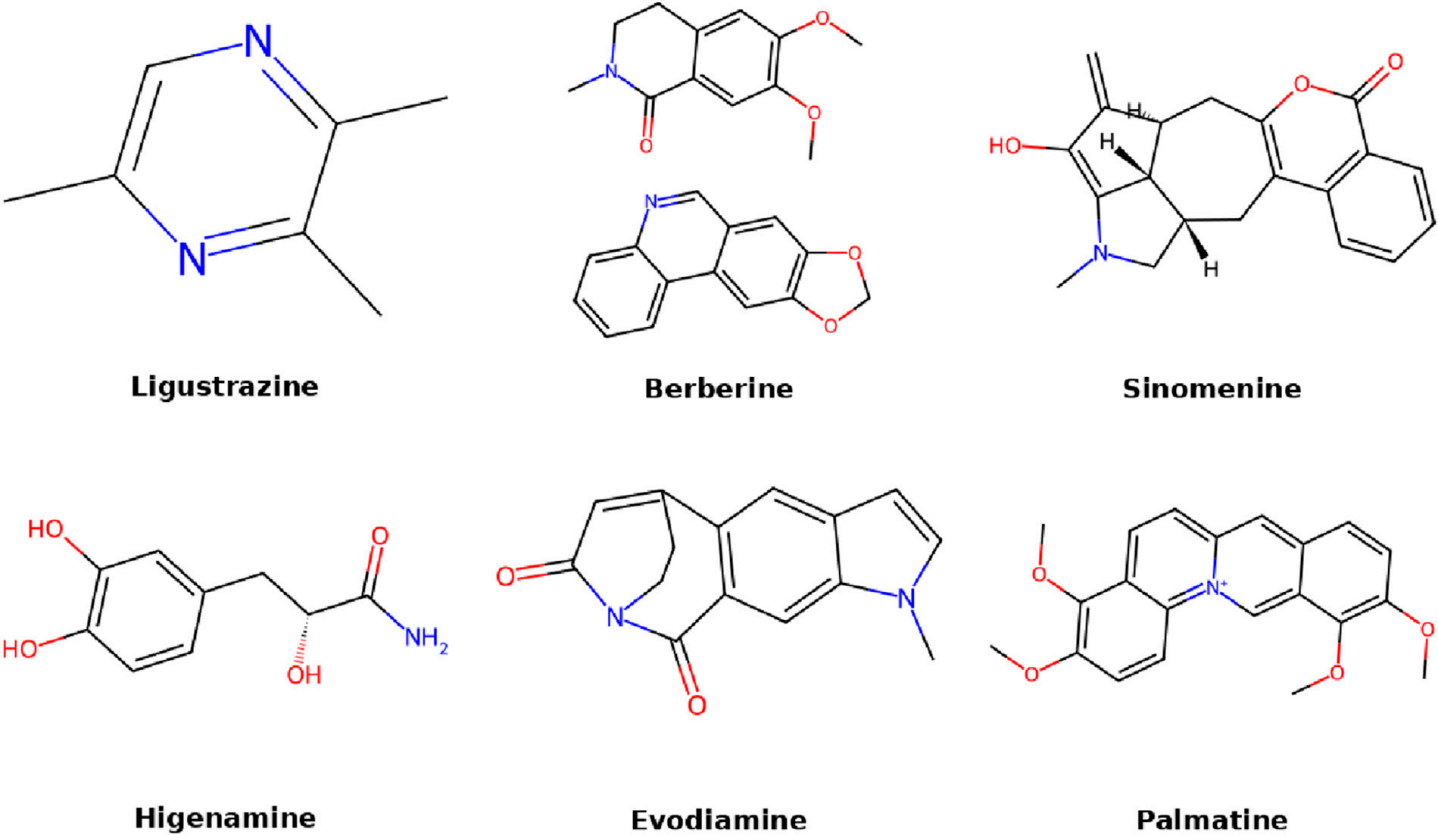
Molecular structures of alkaloids compounds.

Alkaloids are nitrogen-containing compounds with strong pharmacological properties. While structurally distinct from polyphenols, alkaloids also exhibit overlapping anti-inflammatory and anti-apoptotic effects, expanding the functional convergence among diverse natural compounds. In the context of IDD, they have been shown to reduce oxidative stress, suppress inflammatory cytokines, and protect disc cells from apoptosis. Their small molecular size and lipophilicity facilitate cell penetration and biological activity. This section focuses on key alkaloids and their therapeutic roles in IDD (Figure 6).

### 6.1 Ligustrazine

Ligustrazine is a benzopyran-type alkaloid with a tetramethylpyrazine structure, derived from the Umbelliferae plant *Ligusticum chuanxiong*. Its structure is recognized as a clinically effective agent in the treatment of cardiovascular and cerebrovascular diseases (Ma et al., 2024), especially in neuroprotective drug development (Jiang et al., 2025). In rat models of disc degeneration induced by prolonged upright posture, pretreatment with ligustrazine effectively restored disc structure, inhibited the expression of collagen type X, MMP-13, and MMP-3, upregulated type II collagen, and decreased levels of inflammatory factors such as IL-1β, COX-2, and iNOS, showing strong tissue-protective and anti-inflammatory effects, ligustrazine (80 mg/kg) reduced MMP-13 and iNOS ∼60%, improved histological scores by 28%. (Liang et al., 2014).

### 6.2 Berberine

Berberine is an isoquinoline alkaloid widely found in plants of the Berberidaceae family, such as *Coptis chinensis* and *Phellodendron amurense*. Berberine and its derivatives have been proven to treat cardiovascular endothelial injury by regulating endoplasmic reticulum stress, apoptosis, inflammation, oxidative stress, autophagy, platelet dysfunction, and gut microbiota imbalance (Zhang et al., 2025). Berberine also shows therapeutic potential in renal and skin diseases (Fan et al., 2025; A et al., 2025). It reduces TBHP-induced apoptosis in NPCs by activating autophagy, upregulating Bcl-2, and inhibiting pro-apoptotic proteins (Chen et al., 2018). Additionally, it significantly inhibits the expression of MMP-3, MMP-13, and ADAMTS-4/5, reduces ECM degradation, and blocks NF-κB pathway activation, exerting both anti-inflammatory and matrix-protective effects, berberine (10 μM) suppressed cleaved-caspase 3 by 55%, increased Bcl-2 by 1.9-fold, inhibited MMP-13 by 68%. (Lu et al., 2019).

### 6.3 Sinomenine

Sinomenine is a morphinan alkaloid extracted from the Menispermaceae plant *Sinomenium acutum*. It has been used in China as an anti-inflammatory agent for over 30 years (Jiang S. et al., 2024). Sinomenine possesses a wide range of pharmacological effects, including anti-inflammatory, immunosuppressive, antitumor, and neuroprotective activities (Li D. et al., 2023; Li JM. et al., 2023; Jiang P. et al., 2024). It induces autophagy in NPCs, reversing TBHP-induced apoptosis and loss of cell viability; this protective effect is attenuated by the autophagy inhibitor 3-MA, indicating that its mechanism is autophagy-dependent. Animal experiments have also confirmed that sinomenine delays IDD progression (Gao Z. et al., 2019).

### 6.4 Higenamine

Higenamine is a benzylisoquinoline alkaloid and a plant-derived β2-adrenergic receptor agonist. Since 2017, it has been listed on the World Anti-Doping Agency’s prohibited substances list (Rangelov et al., 2022). It possesses antioxidant, anti-apoptotic, anti-inflammatory, electrophysiological regulatory, antifibrotic, and lipid-lowering properties (Chen DT. et al., 2022; Wen et al., 2021; Zhang NN. et al., 2022). Higenamine significantly inhibits IL-1β-induced inflammation in NPCs, reducing the expression of iNOS, COX-2, TNF-α, IL-6, and MMPs (Bai et al., 2019). Moreover, it alleviates apoptosis under inflammatory stimulation by suppressing ROS-mediated PI3K/Akt pathway activation, thus exerting anti-inflammatory and cytoprotective effects (Zhu X. et al., 2021).

### 6.5 Evodiamine

Evodiamine is an indoloquinazoline alkaloid derived from the Rutaceae plant *Evodia rutaecarpa*, and exhibits anticancer, cardioprotective, anti-inflammatory, and anti-Alzheimer’s effects, as well as digestive system protection (Lin et al., 2024; Solanki and Patel, 2024; Zhou et al., 2024). It upregulates SIRT1 expression and activates the PI3K/Akt pathway, effectively inhibiting LPS-induced apoptosis and ECM degradation in human NPCs, reducing MMP-13 and inflammatory cytokines (TNF-α, IL-6), and promoting the synthesis of type II collagen, thereby exerting multiple protective effects (Kuai and Zhang, 2022).

### 6.6 Palmatine

Palmatine is a protoberberine-type isoquinoline alkaloid extracted from the Menispermaceae plant *Fibraurea recisa*. It exhibits protective effects in cardiovascular diseases, osteoporosis, and osteoarthritis (Xin et al., 2024; Li et al., 2023f), which are largely mediated through its antioxidant and anti-inflammatory properties (Ekeuku et al., 2020). Palmatine activates the transcription factor TFEB to enhance autophagy, reduces CHOP expression to alleviate TBHP-induced endoplasmic reticulum stress, and consequently inhibits NPC apoptosis and ECM degradation. Animal studies have shown that palmatine helps preserve disc tissue morphology, indicating its strong potential for structural protection in IDD (Yu et al., 2025).

## 7 Discussion and perspectives

**FIGURE 7:**
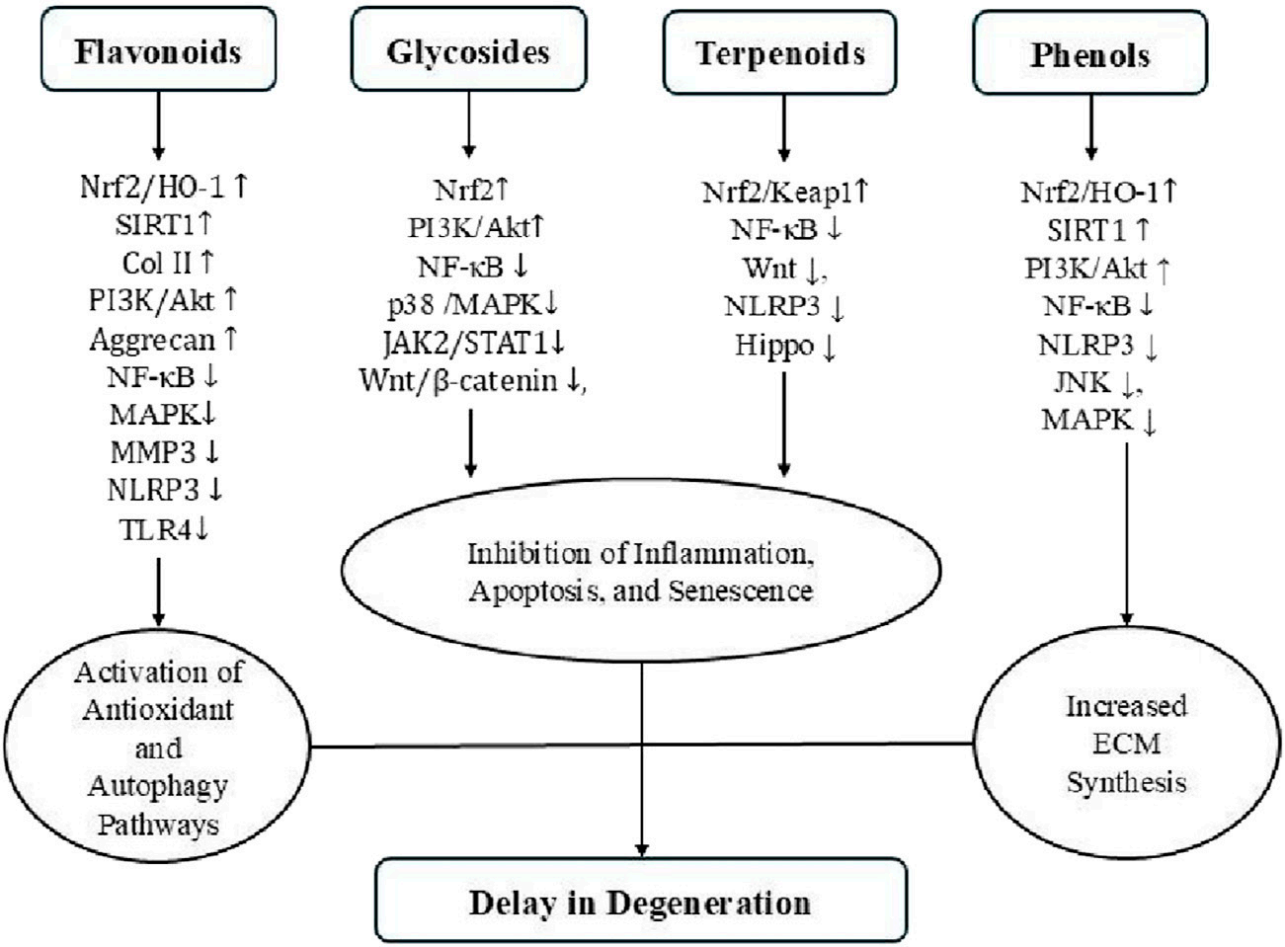
Key signaling pathways and mechanisms of four classes of natural compounds discussed in this review.

Natural products have garnered increasing attention as promising therapeutic candidates for IDD due to their multi-target mechanisms and relatively low toxicity (Sprouffske et al., 2013). The compounds reviewed in this article—spanning flavonoids, glycosides, terpenoids, phenolic compounds, and alkaloids—exert anti-inflammatory, antioxidant, anti-apoptotic, anti-senescent, and ECM-regulating activities, primarily through the modulation of key signaling pathways such as NF-κB, SIRT1, Nrf2, PI3K/Akt, and MAPK (Figure 7) (Chen et al., 2021; Mavrogonatou and Kletsas, 2024). The collective findings provide a compelling preclinical foundation for further development of natural product-based IDD therapies (Table 1).

**TABLE 1:** Key signaling pathways and mechanisms of five classes of natural compounds discussed in this review.

Compound	Source	Signaling pathways	Effects	Key targets
Quercetin	Various fruits and vegetables	p38 MAPK, SIRT1-autophagy, NF-κB, miR-34a/SIRT1	Inhibits apoptosis and ECM degradation, alleviates IDD	p38 MAPK, SIRT1, Keap1–Nrf2, NF-κB
Kaempferol	Tea, broccoli, grapefruit	MAPK, PI3K/Akt, NF-κB	Suppresses inflammation and apoptosis	NF-κB, PI3K/Akt
Luteolin	Celery, green pepper	MAPK, NF-κB	Reduces ECM degradation	MMPs, NF-κB
Apigenin	Parsley, celery	NF-κB, MAPK	Protects NPCs from degeneration	NF-κB, MMPs
Icariin	Epimedium	PI3K/Akt/mTOR	Promotes ECM synthesis	mTOR, LC3
Baicalein	Scutellaria baicalensis	MAPK, NF-κB	Suppresses IL-1β effects	COX-2, MMP-13
Naringin	Citrus fruits	PI3K/Akt, NF-κB	Reduces inflammation and oxidative stress	SOD, NF-κB
Hesperidin	Citrus peel	Nrf2/HO-1	Delays NPC degeneration	Nrf2, HO-1
Genistein	Soy products	ERβ, MAPK	Reduces inflammation and enhances matrix	ERβ, MMPs
Formononetin	Red clover	PI3K/Akt, ERβ	Promotes ECM balance	ERβ, PI3K
Biochanin A	Chickpeas, red clover	NF-κB, MAPK	Delays IDD progression	NF-κB, MMP-13
Ginsenoside Rg1	Panax ginseng	Wnt/β-catenin, YAP/TAZ, NF-κB	Regulates disc homeostasis; inhibits apoptosis, inflammation, ECM degradation	Aggrecan, Collagen II, IL-6, TNF-α, p-p65, IκK
Ginsenoside Rg3	Panax ginseng	p38 MAPK	Inhibits apoptosis, ECM degradation; protects annulus fibrosus	MMP2, MMP3, ADAMTS-4, ADAMTS-5
Notoginsenoside R1 (NGR1)	Panax notoginseng	NF-κB/NLRP3, GSK-3β/β-catenin	Promotes ECM synthesis, inhibits pyroptosis and inflammation	GSK-3β, β-catenin, NLRP3, IL-1β, IL-18
Astragaloside IV (AS-IV)	Astragalus membranaceus	PI3K/Akt, EGFR/MAPK, NF-κB	Reduces inflammation, apoptosis, ECM degradation	IκB-α, NF-κB p65, Bcl-2, Bax, Caspase-3, Col2α1, ADAMTS-5
Dioscin	Dioscorea spp.	MAPK,NF-κB, TLR4	Maintains ECM homeostasis, reduces inflammation	MMP1, MMP3, MMP13, ADAMTS-5, Col2, Aggrecan
Aucubin	Eucommia ulmoides, Aucuba japonica, Plantago asiatica	NF-κB,NLRP3, Wnt/β-catenin	Anti-inflammatory, ECM protection, senescence inhibition	Col2α1, Aggrecan, MMP-13, p-p65 NLRP3
Morroniside	Cornus officinalis	Hippo, Nrf2/Keap1	Reduces senescence, inhibits pyroptosis, protects ECM	p53, p21, MST1/2, LATS1/2, YAP/TAZ
Celastrol	Tripterygium wilfordii	NF-κB	Anti-inflammatory, oxidative stress reduction, ECM protection	MMP-3, MMP-9, MMP-13, ADAMTS-4, COX-2, iNOS
Curcumin	Turmeric	NF-κB, Nrf2, PI3K/Akt	Anti-inflammatory, antioxidant, ECM protection	BDNF, IL-1β, COX-2, TGF-β1/2, MMP-9
Myricetin	Fruits, vegetables, tea, wine	Nrf2/HO-1, NF-κB	Antioxidant, anti-inflammatory, protects NPCs	iNOS, COX-2, MMP-13, ADAMTS-5
Sesamin	Sesame seeds	MAPK, JNK	ECM protection, apoptosis inhibition	CASP3, BAX, BCL2, BECN2, NLRP3
Tea Polyphenols	Green tea	Keap1/Nrf2/ARE	Anti-inflammatory, antioxidant, ECM synthesis	Type II collagen, aggrecan
Resveratrol	Grapes, red wine	SIRT1, NF-κB, PI3K/Akt	SIRT1 activation, anti-senescence	MMP-1, MMP-13, LC3-II, Beclin-1
Proanthocyanidins	Grape seeds	PI3K/Akt, SIRT3/FOXO3, NF-κB	Anti-apoptotic, anti-inflammatory, mitochondrial protection	Bcl-2, Bax, Drp1, OPA1, MFN2
Tyrosol	Olives, fungi	SIRT1, PI3K/Akt, NF-κB/FOXO3	Anti-apoptotic, anti-inflammatory	SIRT1, PGE2, TNF-α, IL-6
Ligustrazine	Ligusticum chuanxiong	NF-κB, COX-2, iNOS	Anti-inflammatory, tissue-protective	MMP-13, iNOS; Col2
Berberine	Coptis chinensis, Phellodendron amurense	NF-κB, Bcl-2, Caspases	Anti-apoptotic, anti-inflammatory	MMP-3, MMP-13, Bcl-2
Sinomenine	Sinomenium acutum	Autophagy pathway	Anti-inflammatory, autophagy-inducing	Autophagy markers
Higenamine	Various plants	PI3K/Akt, ROS	Anti-apoptotic, anti-inflammatory	iNOS, COX-2, TNF-α
Evodiamine	Evodia rutaecarpa	SIRT1, PI3K/Akt	Anti-apoptotic, anti-inflammatory	MMP-13, TNF-α; Col2
Palmatine	Fibraurea recisa	TFEB, CHOP	Anti-inflammatory, antioxidant	Autophagy, CHOP

Despite significant progress in basic and animal research, the clinical translation of natural products remains limited. Most studies are confined to *in vitro* models or small animal experiments, with a notable lack of human clinical trials directly targeting IDD. For instance, compounds such as curcumin, resveratrol, and quercetin have been assessed in clinical trials for osteoarthritis or systemic inflammation, but no large-scale clinical investigations have addressed their efficacy in IDD (Novais et al., 2021; Ataei et al., 2023; Kwon). Moreover, inconsistencies in dosage, treatment duration, and outcome measures impede direct comparisons and evidence-based application.

Several obstacles hinder the successful translation of natural products from bench to bedside. First, many compounds suffer from poor oral bioavailability, rapid metabolism, and low accumulation within the avascular intervertebral disc environment (Szabó et al., 2022; Araya-Sib et al., 2021). Second, physiological differences between rodent models and the human spine limit the extrapolation of pharmacological responses (Liu Z. et al., 2023). Third, standardized delivery platforms and scalable production processes remain underdeveloped. Addressing these limitations is crucial for advancing natural compounds into clinical practice.

Recent advances in biomaterials have introduced innovative delivery strategies to improve the stability, targeting, and bioactivity of natural products. Notably, nanoparticle encapsulation, thermosensitive hydrogels, and injectable biocompatible gels have demonstrated enhanced efficacy in preclinical IDD models (Ike et al., 2022; Shi S. et al., 2024). For example, kaempferol-loaded fibrin hydrogels and PLGA-PEG-PLGA-based resveratrol systems have shown sustained release profiles and regenerative potential in rat models (Gao et al., 2023; Liu et al., 2025). However, long-term safety, degradation kinetics, and regulatory pathways for these systems remain largely unverified in humans.

To overcome existing challenges, future studies should prioritize:rigorous pharmacokinetic and pharmacodynamic evaluations in large-animal models. Early-phase clinical trials targeting IDD-specific patient populations. Combination strategies that integrate multiple natural compounds or adjunctive use with conventional treatments (Mavrogonatou and Kletsas, 2024). The development of modular and customizable drug delivery platforms. Furthermore, the integration of computational modeling, high-throughput screening, and network pharmacology may accelerate the identification of synergistic interactions and optimize formulation strategies (Sun W. et al., 2022; Wang X. et al., 2023; Liu H. et al., 2023).

In conclusion, natural products represent a rich and underutilized resource for IDD therapy. With coordinated efforts across pharmacology, materials science, and clinical research, their full therapeutic potential can be more effectively translated into viable interventions for degenerative spinal disorders (Table 2).

**TABLE 2:** Structure-activity relationship of natural compounds in IDD.

Compound	Class	Key structural features	Reported activities in IDD	Structure–Activity notes
Hyperoside	Flavonoid	Quercetin-3-O-galactoside	Anti-inflammatory, ECM protection	Glycosylation enhances water solubility and cellular uptake
Quercetin	Flavonoid	3′,4′-dihydroxy B ring, 3-OH on C ring	Antioxidant, anti-inflammatory, pro-autophagy	Catechol enhances NF-κB inhibition and radical scavenging
Apigenin	Flavonoid	5,7-dihydroxy A ring, 4′-OH B ring	Senescence delay, autophagy induction	Hydroxyl pattern modulates AMPK pathway
Butein	Flavonoid	3,4-dihydroxy B ring, 4′-OH	SIRT1 activation, reduces oxidative stress	Polyphenolic structure linked to SIRT1 modulation
Baicalein	Flavonoid	5,6,7-trihydroxy A ring	Inhibits COX-2, promotes ECM gene expression	Trihydroxy enhances ROS neutralization
Kaempferol	Flavonoid	4′-OH, 3-OH, no catechol B ring	Anti-oxidative, ECM metabolism balance	Fewer hydroxyls = weaker anti-inflammatory vs quercetin
Luteolin	Flavonoid	3′,4′-dihydroxy B ring, 5,7-dihydroxy A ring	Anti-apoptotic, Nrf2/HO-1 activation	Catechol B ring supports ROS scavenging
Naringin	Flavonoid	Flavanone glycoside, 4′-OH	Reduces IL-1β, protects NPCs	Glycoside enhances bioavailability
Pinocembrin	Flavonoid	5,7-dihydroxyflavanone	Anti-inflammatory, mitochondrial protection	Flavanone skeleton stabilizes ROS response
Icariin	Flavonoid	Prenylated flavonoid with glycoside	Activates autophagy, ECM protection	Prenyl group enhances lipophilicity and bioactivity
Fisetin	Flavonoid	3,7,3′,4′-tetrahydroxyflavone	Reduces oxidative damage, promotes chondrogenesis	Hydroxylation key to antioxidant capacity
Acacetin	Flavonoid	5,7-dihydroxy-4′-methoxyflavone	NF-κB inhibition, ECM homeostasis	Methoxy substitution modulates lipophilicity
Ginsenoside Rg1	Glycoside	Steroid backbone with sugar moieties	ECM upregulation, inflammation inhibition	Glucose moieties promote ECM stimulation
Ginsenoside Rg3	Glycoside	Steroid glycoside with fewer sugar units	Anti-apoptotic, inhibits inflammatory cytokines	Fewer sugars may enhance permeability
Notoginsenoside R1	Glycoside	Dammarane-type saponin	Inhibits TNF-α, MMPs, enhances ECM	High glycosylation enhances solubility
Astragaloside IV	Glycoside	Cycloartane-type triterpenoid glycoside	Reduces oxidative stress, inhibits apoptosis	Sugar groups modulate transport
Diosgenin	Glycoside	Spirostan-type sapogenin	NF-κB pathway inhibition	Steroid-like core enhances membrane activity
Kinsenoside	Glycoside	Lactone ring with sugar unit	Reduces ROS, prevents apoptosis	Lactone may activate survival pathways
Crocin	Glycoside	Polyene chain with sugar ends	MMP suppression, anti-inflammatory	Glycosylation aids in cytokine modulation
Loganin	Terpenoid	Iridoid glycoside	Anti-apoptosis, ECM protection	Iridoid core supports anabolic signaling
Morroniside	Terpenoid	Secoiridoid structure with glucose	Anti-inflammatory, collagen synthesis	Iridoid ring promotes matrix balance
Celastrol	Terpenoid	Quinone methide triterpenoid	MMP-13 inhibition, MRI signal increase	Quinone group inhibits NF-κB and proteases
Curcumin	Phenol	β-diketone with phenolic rings	Anti-inflammatory, neuroprotective	Conjugated system suppresses p65 activity
Myricetin	Phenol	Polyhydroxylated flavonol	ROS inhibition, ECM upregulation	Hydroxyl groups support antioxidant effect
Sesamin	Phenol	Lignan with methylenedioxy groups	Reduces TNF-α, IL-6 expression	Lignan backbone interacts with inflammation genes
Tea Polyphenols	Phenol	EGCG, EGC *etc.*, gallate esters	Inhibits IL-1β, protects ECM	Galloyl moieties chelate ROS agents
Resveratrol	Phenol	Stilbene with 3,5,4′-OH	Autophagy induction, apoptosis inhibition	Planar ring system supports SIRT1 and Beclin activation
Proanthocyanidins	Phenol	Condensed tannin polymers	Antioxidant, matrix protection	Polymer size affects bioavailability
Tyrosol	Phenol	Phenylethanol structure	Anti-inflammatory, cytoprotection	Simple phenol supports cell viability
Gingerol	Phenol	Alkyl chain with phenol and hydroxyl	IL-6, TNF-α inhibition, ECM balance	Alkyl tail aids in membrane interaction
Berberine	Alkaloid	Isoquinoline alkaloid with quaternary ammonium structure	Suppresses inflammation, enhances autophagy, inhibits ECM degradation	Quaternary ammonium structure promotes cell entry and NF-κB inhibition
Ligustrazine	Alkaloid	Tetramethylpyrazine core	Improves microcirculation, reduces apoptosis, inhibits p38/MAPK	Small molecular size enhances tissue penetration and anti-inflammatory activity
Sinomenine	Alkaloid	Morphinan-type alkaloid	Induces autophagy, reduces TBHP-induced apoptosis, delays IDD progression	Morphinan structure supports autophagy-dependent protective effects
Higenamine	Alkaloid	Benzylisoquinoline alkaloid, β2-adrenergic agonist	Suppresses inflammation, reduces apoptosis, inhibits ROS-mediated PI3K/Akt pathway	Aromatic backbone modulates inflammation and β2 signaling
Evodiamine	Alkaloid	Indoloquinazoline scaffold	Activates SIRT1/PI3K-Akt, inhibits apoptosis and ECM degradation, promotes Col II	Indole and quinazoline units contribute to multifunctional bioactivity
Palmatine	Alkaloid	Protoberberine-type isoquinoline	Activates TFEB, relieves ER stress, reduces NPC apoptosis and ECM loss	Protoberberine framework facilitates autophagy and ER homeostasis

## 8 Conclusion

Natural products regulate multiple critical signaling pathways and exhibit significant anti-inflammatory, antioxidant, anti-apoptotic, and regenerative effects, suggesting their potential to delay or even reverse the pathological progression of IDD. Although research into natural product-based interventions for IDD remains in the preclinical phase, their potential for clinical translation is increasingly recognized. Future studies should focus on optimizing drug delivery systems and exploring combination therapies to facilitate the efficient translation of natural product-based interventions from basic research to clinical application.

It is important to acknowledge that several plant-derived metabolites included in this review—such as flavonoids and polyphenols—are known to fall within the category of pan-assay interference compounds, particularly in in vitro assays. These compounds may produce misleading pharmacological signals by non-specifically interacting with a variety of targets or assay components. Therefore, while this review summarizes mechanistic studies, emphasis was placed on *in vivo* findings to reduce the overinterpretation of results from PAINS-prone compounds. Further studies using orthogonal assays, target deconvolution, and structure–activity analyses are needed to validate their pharmacological relevance.
